# Integrated analysis of colorectal cancer metastasis identifies characteristics of tumor cell during metastasis

**DOI:** 10.1093/gastro/goae055

**Published:** 2024-05-30

**Authors:** Haoyu Fu, Xiaohuan Lu, Tiantian Ji, Liping Wang, Guobin Wang, Lin Wang, Zheng Wang

**Affiliations:** Department of Gastrointestinal Surgery, Union Hospital, Tongji Medical College, Huazhong University of Science and Technology, Wuhan, Hubei, P. R. China; Research Center for Tissue Engineering and Regenerative Medicine, Union Hospital, Tongji Medical College, Huazhong University of Science and Technology, Wuhan, Hubei, P. R. China; Hubei Key Laboratory of Regenerative Medicine and Multi-disciplinary Translational Research, Wuhan, Hubei, P. R. China; Hubei Provincial Engineering Research Center of Clinical Laboratory and Active Health Smart Equipment, Wuhan, Hubei, P. R. China; Department of Gastrointestinal Surgery, Union Hospital, Tongji Medical College, Huazhong University of Science and Technology, Wuhan, Hubei, P. R. China; Research Center for Tissue Engineering and Regenerative Medicine, Union Hospital, Tongji Medical College, Huazhong University of Science and Technology, Wuhan, Hubei, P. R. China; Hubei Key Laboratory of Regenerative Medicine and Multi-disciplinary Translational Research, Wuhan, Hubei, P. R. China; Hubei Provincial Engineering Research Center of Clinical Laboratory and Active Health Smart Equipment, Wuhan, Hubei, P. R. China; Research Center for Tissue Engineering and Regenerative Medicine, Union Hospital, Tongji Medical College, Huazhong University of Science and Technology, Wuhan, Hubei, P. R. China; Hubei Key Laboratory of Regenerative Medicine and Multi-disciplinary Translational Research, Wuhan, Hubei, P. R. China; Hubei Provincial Engineering Research Center of Clinical Laboratory and Active Health Smart Equipment, Wuhan, Hubei, P. R. China; Research Center for Tissue Engineering and Regenerative Medicine, Union Hospital, Tongji Medical College, Huazhong University of Science and Technology, Wuhan, Hubei, P. R. China; Hubei Key Laboratory of Regenerative Medicine and Multi-disciplinary Translational Research, Wuhan, Hubei, P. R. China; Hubei Provincial Engineering Research Center of Clinical Laboratory and Active Health Smart Equipment, Wuhan, Hubei, P. R. China; Department of Clinical Laboratory, Union Hospital, Tongji Medical College, Huazhong University of Science and Technology, Wuhan, Hubei, P. R. Chin; Department of Gastrointestinal Surgery, Union Hospital, Tongji Medical College, Huazhong University of Science and Technology, Wuhan, Hubei, P. R. China; Research Center for Tissue Engineering and Regenerative Medicine, Union Hospital, Tongji Medical College, Huazhong University of Science and Technology, Wuhan, Hubei, P. R. China; Hubei Key Laboratory of Regenerative Medicine and Multi-disciplinary Translational Research, Wuhan, Hubei, P. R. China; Hubei Provincial Engineering Research Center of Clinical Laboratory and Active Health Smart Equipment, Wuhan, Hubei, P. R. China; Department of Clinical Laboratory, Union Hospital, Tongji Medical College, Huazhong University of Science and Technology, Wuhan, Hubei, P. R. Chin; Department of Gastrointestinal Surgery, Union Hospital, Tongji Medical College, Huazhong University of Science and Technology, Wuhan, Hubei, P. R. China; Research Center for Tissue Engineering and Regenerative Medicine, Union Hospital, Tongji Medical College, Huazhong University of Science and Technology, Wuhan, Hubei, P. R. China; Hubei Key Laboratory of Regenerative Medicine and Multi-disciplinary Translational Research, Wuhan, Hubei, P. R. China; Hubei Provincial Engineering Research Center of Clinical Laboratory and Active Health Smart Equipment, Wuhan, Hubei, P. R. China

**Keywords:** colorectal cancer, metastasis, single-cell RNA sequencing

## Abstract

**Background:**

Metastasis is the main cause of death in colorectal cancer (CRC). Metastasis is a sequential and dynamic process, but the development of tumor cells during this process is unclear. In this study, we aimed to reveal characteristics of tumor cell subset during CRC metastasis.

**Methods:**

Single-cell RNA sequence CRC data of normal epithelium, non-metastatic primary tumor, metastatic primary tumor, and liver metastases from gene expression omnibus (GEO) dataset were analyzed to reveal characteristics of CRC metastasis. Primary tumor tissues of three non-metastatic CRC and three metastatic CRC patients from Union Hospital of Tongji Medical College (Wuhan, China) were used to verify the characteristics of CRC metastasis.

**Results:**

We identified a metastasis-related cancer cell subset EP1, which was characterized with a high expression of *KRT17*, *LAMC2*, *EMP1*, and *PLAC8*. EP1 had an enhanced cell–cell interaction, which interacted with *SPP*^+^ macrophages and drove them toward anti-inflammatory and immunosuppressive phenotype. Dynamic changes in genes and TF regulons during the metastasis were also revealed.

**Conclusions:**

This study advanced our understanding of the development of tumor cells during CRC metastasis and further identified metastasis-related subset and potential therapeutic targets for the treatment and prevention of CRC metastasis.

## Introduction

Colorectal cancer (CRC) ranks third in incidence and second in mortality among cancer globally [1]. The main cause of death in CRC patients is metastasis, with the liver being the most frequently metastatic site. Approximately 20% of CRC patients are diagnosed with metastatic CRC and show a poor prognosis with 5-year survival lower than 20% [2]. Therefore, researches on revealing characteristics of CRC metastasis and finding new therapeutic strategies are highly important to improve CRC prognosis.

Metastasis is a complex process involving cancer cells and tumor microenvironment. During cancer metastasis, cancer cells go through primary tumor, local invasion, spreading to circulation, and colonizing the distal secondary sites [3]. Phenotypes of cancer cell dynamically alter during the process. Cancer cells have the ability to proliferate in metastasis initiation [4]; however, they exhibit epithelial-to-mesenchymal transition features during invasion [5]. When reaching the metastasis, cancer cells enter dormancy or recover proliferation [6, 7]. Immunosuppressive tumor microenvironment also plays an important role in metastasis [8]. Myeloid-derived suppressor cells and T-regulatory cells can promote metastasis by suppressing tumor-specific antigen presentation, permitting tumor cells to escape from immune killing [9–11]. Hypoxia also supports tumor progression and metastasis through hypoxia-inducible factor 1, including regulation of tumor angiogenesis, the metabolism of glucose, and extracellular matrix remodeling [12, 13]. Current research mainly focuses on the role of gene or cell in a certain stage of metastasis, but less on the whole process of metastasis. Liver metastasis typically occurs at an advanced stage of CRC, involving the detachment of tumor cells from the primary tumor, invasion into surrounding tissues or vessels, migration, and ultimately colonization of the liver. However, the dynamic changes of cancer cells in this metastasis process are still unclear.

In this study, we analyzed single-cell RNA sequence data of normal epithelium (NE), primary tumor of non-metastatic CRC (NMPT), primary tumor of metastatic CRC (MPT), and liver metastases (LM) of metastatic CRC from GEO dataset and identified a metastasis-related cancer cell subset named EP1. EP1 can adhere to vascular endothelium and promote *SPP*^+^ macrophages, a newly identified macrophage subset associated with worse prognosis [14, 15], into an anti-inflammatory and immunosuppressive phenotype. Additionally, we elucidated the evolutionary characteristics of cancer cells during metastasis and found some potential therapeutic targets. Our research identified characteristics of CRC metastasis and provided a new idea for the prediction and treatment of metastatic CRC.

## Materials and methods

### Human specimens

Primary tumor tissues of three non-metastatic CRC and three metastatic CRC patients were collected from the Department of Gastrointestinal Surgery, Union Hospital of Tongji Medical College, Huazhong University of Science and Technology, Wuhan, China, between July 2023 and February 2024. Specimens were taken directly from the tumor. The clinical information of these patients is listed in Supplementary Table S1. The study conformed with the principles outlined in the Declaration of Helsinki and obtained ethical approval from the Institutional Review Board of Tongji Medical College (permission number: S002).

### Cell lines

CRC cell line HCT116 (RRID: CVCL_0291) was purchased from American Type Culture Collection (Manassas, USA). All experiments were performed with mycoplasma-free cells.

### Data processing

Single-cell sequencing data (GSE221575) and spatial transcriptomics data (GSE221575 and GSE225857) were obtained from the GEO database. The transcriptome data and clinical information of the cancer genome atlas (TCGA) CRC patients were obtained from UCSC Xena (http://xena.ucsc.edu/). FPKM data of TCGA COAD and READ were used for subsequent analyses. The single-cell sequencing data were converted to a Seurat object using R package Seurat (version 4.3.0). To filter out low-quality cells, cells with features <200 or >6,000 or mitochondrial genes >20% were excluded. Then cells were annotated using R package SingleR (version 1.8.1) [16]. Tumor cells and normal cells in epithelial cells were identified using R package SCEVAN (version 1.0.1). Mitochondrial and ribosomal genes were excluded from the analysis. We used a Seurat approach to remove batch effects. With FindIntegrationAnchors function, we created anchors using top 2,000 high variable genes. Then, IntegrateData function was used to create an integrated matrix without batch effect. Dimensionality reduction was performed through principal component analysis. With resolution = 0.5, nine clusters were identified using FindClusters function and were visualized with two-dimensional UMAP plots.

### Functional enrichment analysis

To explore the function of different clusters, differential genes were identified by FindMarkers function provided by R package Seurat. Gene ontology (GO) and gene set enrichment analysis (GSEA) were performed using R package clusterProfiler (version 4.2.2), and gene set variation analysis (GSVA) was conducted using R package GSVA (version 1.42.0). Gene sets were obtained from MSigDB (https://www.gsea-msigdb.org/).

### Evaluation of specific types of cells in tumor tissue

CYBERSORTx [17] was applied to evaluate the abundance of cell subsets in tumor tissue. We constructed a signature matrix using single-cell sequencing data and imputed cell fractions using default parameters.

### Cell–cell communication analysis

Cell–cell communication analysis was performed using CellPhoneDB (version 3.0.0). CellPhoneDB is a tool to study cell–cell communication from single-cell transcriptomics data based on Python. The interactions of 12 kinds of cells of a total of 7,560 cells were evaluated.

### siRNA transfection

Expression level of *EMP1* in CRC cell line HCT116 was knocked down using siRNA. HCT116 cells were transfected with *EMP1* siRNA or control siRNA using Lipofectamine RNAiMAX (Invitrogen) for 48 h. *EMP1* siRNA and control siRNA were ordered from RiboBio (Guangzhou, China).

### Transwell migration assay

Transwell migration assay was performed to evaluate the migration ability of HCT116. Transwell chambers were placed in a 24-well plate, then 200 μL of serum-free medium with 5 × 10^4^ cells was added to the upper chamber, and 700 μL of medium with 10% serum was added to the lower chamber. After 24-h culture, the transwell chambers with cells were fixed with 4% paraformaldehyde and then stained with 0.1% crystal violet. The migrating cells were imaged under a microscope and the number of cells was counted in five non-repeating fields.

### Immunofluorescence

Immunofluorescence staining of CRC tumor tissues was performed according to standard protocol. Following antigen retrieval and autofluorescence quenching, the tissue sections were incubated with bovine serum albumin for a duration of 30 min. Then, tissue sections were incubated with rabbit anti-human *CD14* (Abcam, ab133335), rabbit anti-human *SPP1* (Proteintech, 22952–1-AP), and rabbit anti-human *LAMC2* (Abclonal, A1869) overnight. Tissue sections were washed three times with PBS and incubated with secondary fluorescent antibodies for 50 min. Subsequently, the nuclei were counterstained with DAPI. The fluorescence microscope was used for visualization.

### Trajectory analysis

To explore the evolution characteristics of metastatic CRC cells, we performed pseudotime analysis and gene regulatory network analysis. Pseudotime analysis was performed using the R package Monocle3 (version 1.3.1). The UMI matrix was converted into a CellDataSet object. We used the align_cds function to remove the batch effect. Trajectory graph was built using the function learn_graph. Gene regulatory network analysis was performed using R package SCENIC (version 1.3.0). The area under the curve score of each TF regulons in each cell was calculated following the SCENIC tutorial.

### Quantitative real-time polymerase chain reaction (qRT-PCR)

Total RNA was extracted from cells by the RNA-easy Isolation Reagent (Vazyme, R701-02). cDNA synthesized from 12 μg of total RNA with the M-MLV (H-) Reverse Transcriptase System (Vazyme, R021-01) was subjected to RT-qPCR by the AceQ qPCR SYBR Green Master Mix (Vazyme, Q111-02). The analysis of target gene expression was followed by the 2^–ΔΔCT^ method. The specific primers are listed in Supplementary Table S2.

### Statistical analysis

Statistical analysis was performed using R Statistical Software and GraphPad Prism software. The results are presented as means ± SD or SEM. Student’s *t* test was applied for statistical analysis. A *P* value of <0.05 indicated a significant difference.

## Results

### Single-cell RNA sequencing reveals heterogeneity of the metastatic CRC cells

**Figure 1.**
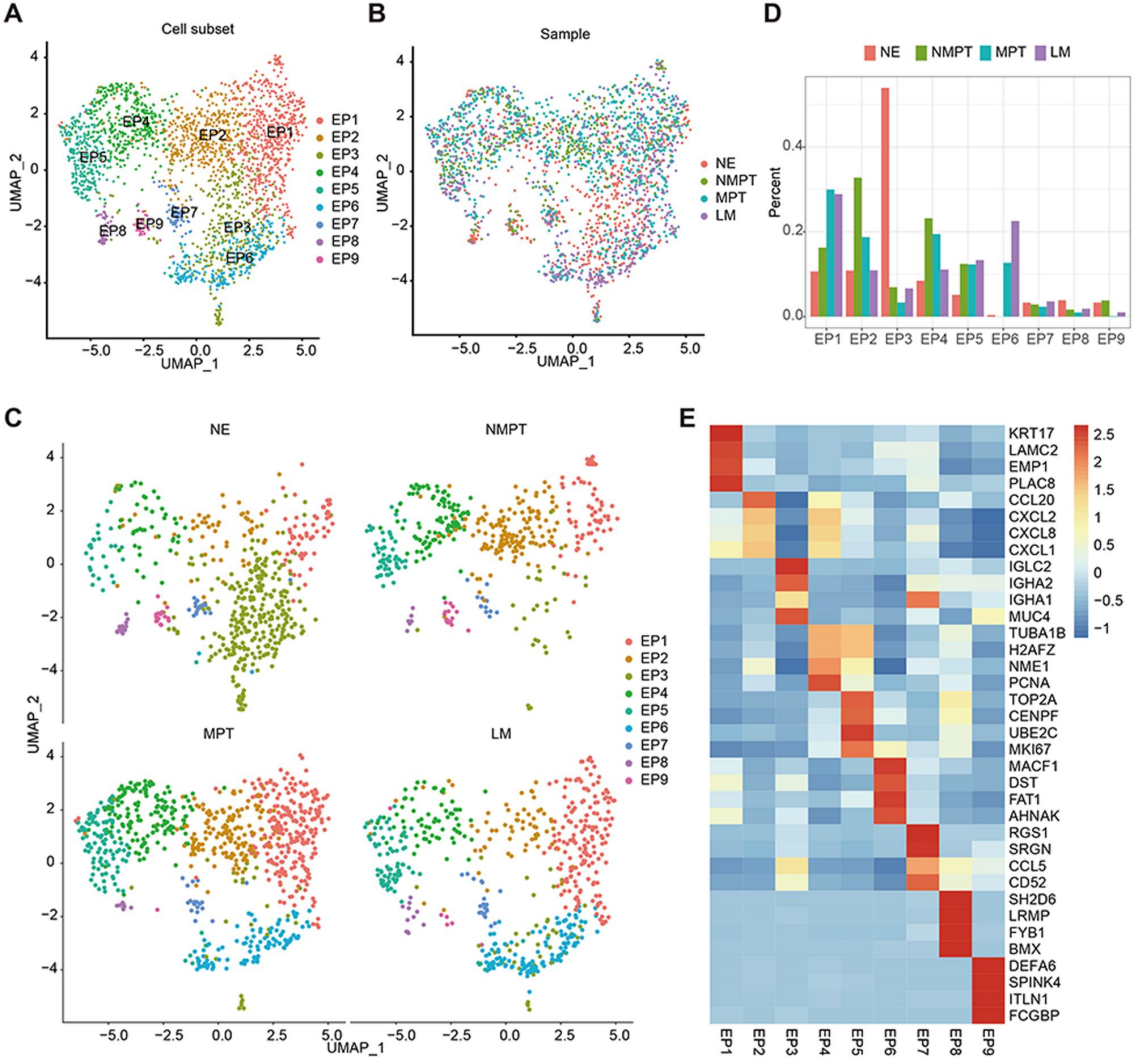
Identification of tumor cell subsets in metastatic colorectal cancer. (A) Uniform manifold approximation and projection (UMAP) plot of tumor cells, cells were colored by cell subsets. (B, C) UAMP plot of tumor cells (B), cells were colored by sample. Box plot showed proportion of different samples in different clusters (C). (D) Split UMAP plot showed proportion of different cell subsets in different samples. (E) Heatmap showed marker genes of cell subsets.

We analyzed the single-cell sequencing data of NE, NMPT, MPT, and LM. After quality control and cell annotation, the epithelial cell was selected for further analysis (Supplementary Figure S1A). Malignant tumor cells were identified by copy number variation (CNV) analysis (Supplementary Figure S1B). Normal epithelial cells and malignant tumor cells were clustered into nine cell subsets (EP1, EP2, EP3, EP4, EP5, EP6, EP7, EP8, EP9; Figure 1A). Proportions of cell subsets in the different samples showed highly significant heterogeneity (Figure 1B, C). EP1 had high expression levels of *KRT17*, *LAMC2*, *EMP1*, and *PLAC8*. The proportion of EP1 was low in NMPT while high in MPT and LM. These results suggested EP1 could be a metastasis-related cell subset. EP2 showed a high expression of chemokines genes (*CCL20*, *CXCL1*, *CXCL2*, and *CXCL8*), and high proportion in NMPT while low proportion in MPT and LM. EP3 was characterized by high expression of immunoglobulin-related genes (*IGLC2*, *IGHA1*, *IGHA2*) and *MUC4*. This cell subset was predominantly present in NE and was identified as cell subset of normal epithelial cells. EP5 highly expressed cell cycle-related genes (*TOP2A*, *CENPF*, *UBE2C*, and *MKI67*), suggesting that EP5 is a proliferating cell subset. A high expression of *MACF1*, *DST*, *FAT1*, and *AHNAK* was found in EP6. EP6 was mainly present in the MPT and LM (Figure 1D, E; Supplementary Figure S1C). These results demonstrated a significant heterogeneity in metastatic CRC cells.

### EP1 was associated with CRC metastasis

**Figure 2.**
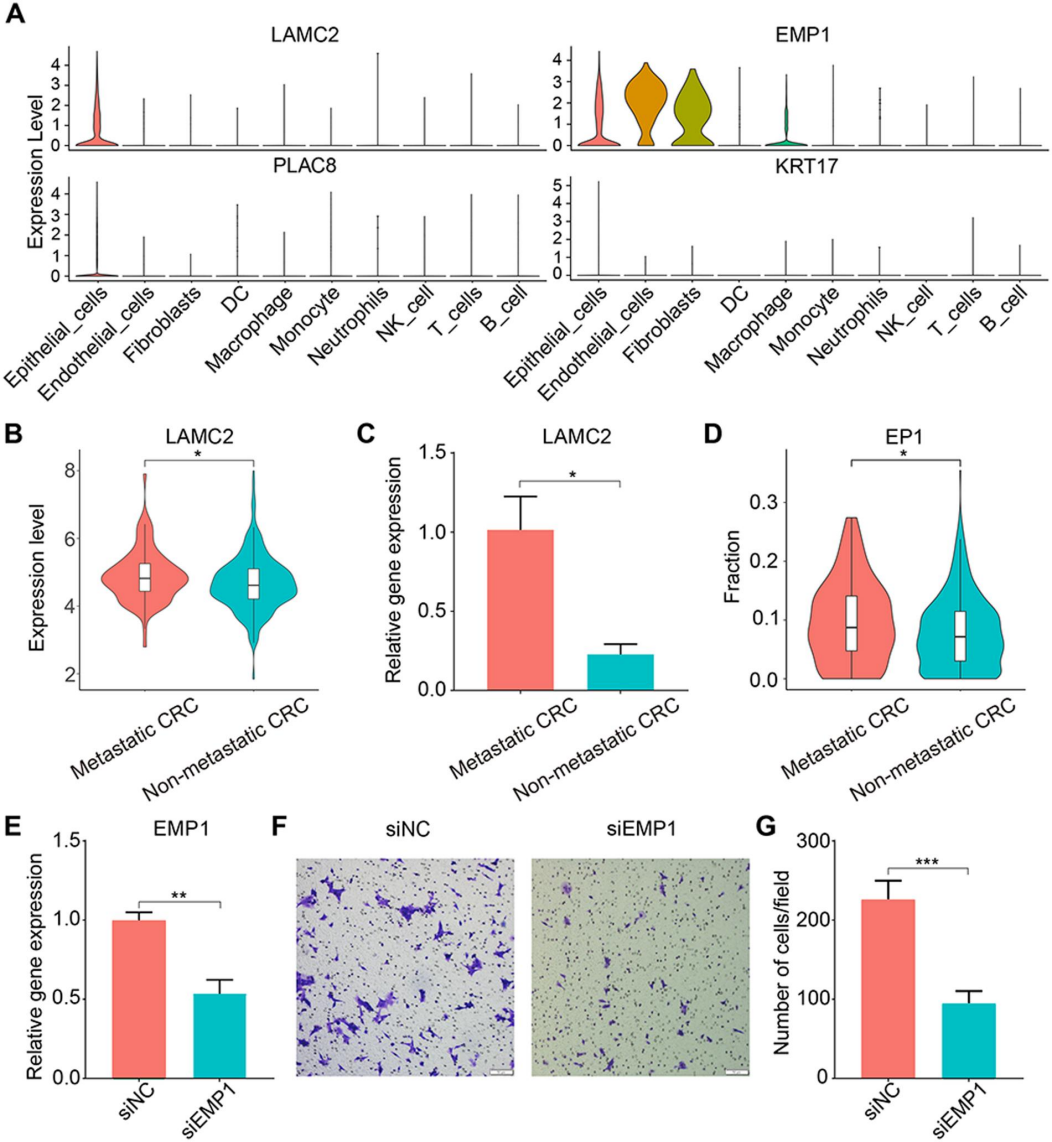
EP1 was a metastasis-related cell subset. (A) Expression level of *LAMC2*, *EMP1*, *PLAC8*, and *KRT17* in different types of cells. (B) Expression level of *LAMC2* in the cancer genome atlas (TCGA) metastatic and non-metastatic colorectal cancer (CRC) patients. (C) Quantitative real-time polymerase chain reaction (qRT-PCR) showed the relative gene expression of *LAMC2* in metastatic and non-metastatic CRC patients. (D) Abundance of EP1 in TCGA metastatic and non-metastatic CRC patients. (E) qRT-PCR showed the relative gene expression of *EMP1* in HCT116 after control siRNA (siNC) and *EMP1* siRNA (siEMP1) transfection. (F) Transwell migration assay of siNC and siEMP1. (G) Bar plot showed migrating cells of siNC and siEMP1. Scale bars, 50 μm. *P value < 0.05, **P value < 0.01, ***P value < 0.001.

EP1 was mainly represented in metastasis-related tissue MPT and LM, suggesting its role in CRC metastasis. To verify the finding, we analyzed the expression level of marker genes of EP1 (*LAMC2*, *EMP1*, *PLAC8*, *KRT17*) in different types of cells. Results showed that *LAMC2* was highly expressed only in epithelial cells (Figure 2A) and represented the abundance of EP1 in CRC tissues. Analysis of expression level of *LAMC2* in TCGA CRC samples showed metastatic CRC patients had a higher expression level of *LAMC2* than non-metastatic CRC patients (Figure 2B). qRT-PCR of tumor tissues from metastatic and non-metastatic CRC patients also showed *LAMC2* is higher expressed in metastatic CRC patients than in non-metastatic CRC patients (Figure 2C). Abundance of EP1 in tumor tissue was further evaluated with CYBERSORTx, and results also showed a higher abundance in metastatic CRC patients than in non-metastatic CRC patients (Figure 2D). To further verify the relationship between marker genes of EP1 and metastasis, *EMP1* in CRC cell line HCT116 was knocked down through siRNA (Figure 2E). Cell migration experiment showed that cell migration ability is reduced after knocking down (*EMP1)* (Figure 2F, G). These results indicated that EP1 was a metastasis-related cell subset.

### Metastasis-related cell subset EP1 was characterized with enhanced cell–cell interaction

EP1 and EP6 were mainly represented in metastasis-related tissue MPT and LM. We explored the function signatures of the metastasis-related cell subsets EP1 and EP6. GO enrichment analysis showed an enrichment of actin filament organization and microtubule-based movement process in the EP6 (Supplementary Figure S2A). GSEA revealed a significant enrichment of the RhoA GTPase Cycle pathway in the EP6 (Supplementary Figure S2B).

**Figure 3.**
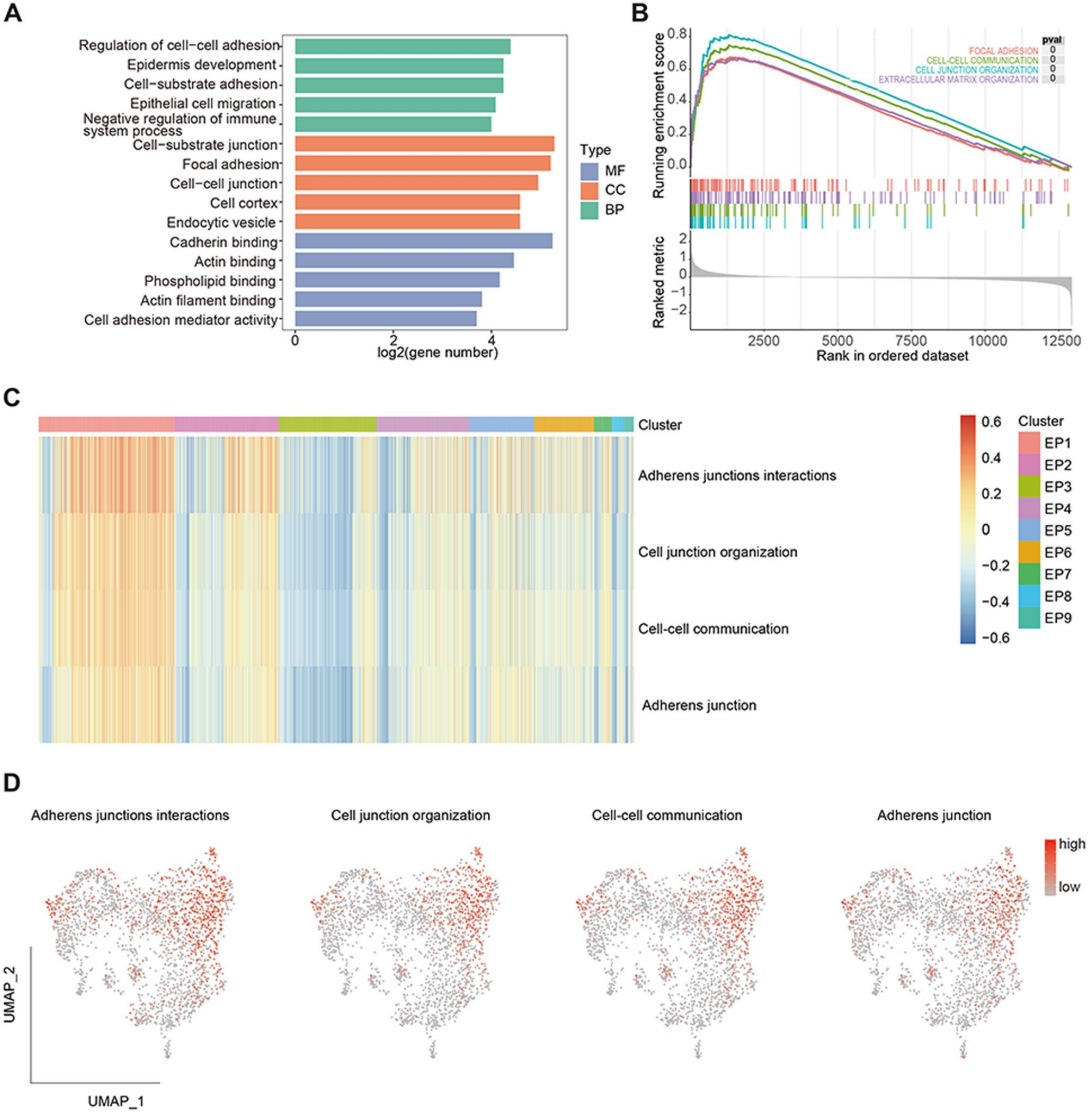
Functional enrichment analysis of metastasis-related cell subset EP1. (A). Gene ontology analysis of EP1, bar plot showed enrichment of biological process (BP), cellular component (CC), and molecular function (MF) in EP1. (B) Gene set enrichment analysis of EP1. Heatmap (C) and UMAP plot (D) showed score of different cell subsets.

For metastasis-related cell subset EP1, GO enrichment analysis showed significant enrichment of cell–substrate adhesion and negative regulation of immune system process (Figure 3A). GSEA analysis showed the enrichment of focal adhesion and cell–cell communication pathway (Figure 3B). Furthermore, GSVA showed enhanced cell–cell interactions in EP1 (Figure 3C, D). These results indicate the enhanced cell–cell interaction functions in metastasis-related cell subset EP1.

### The enhanced cell–cell interactions between the EP1 and *SPP*^+^ macrophage

**Figure 4.**
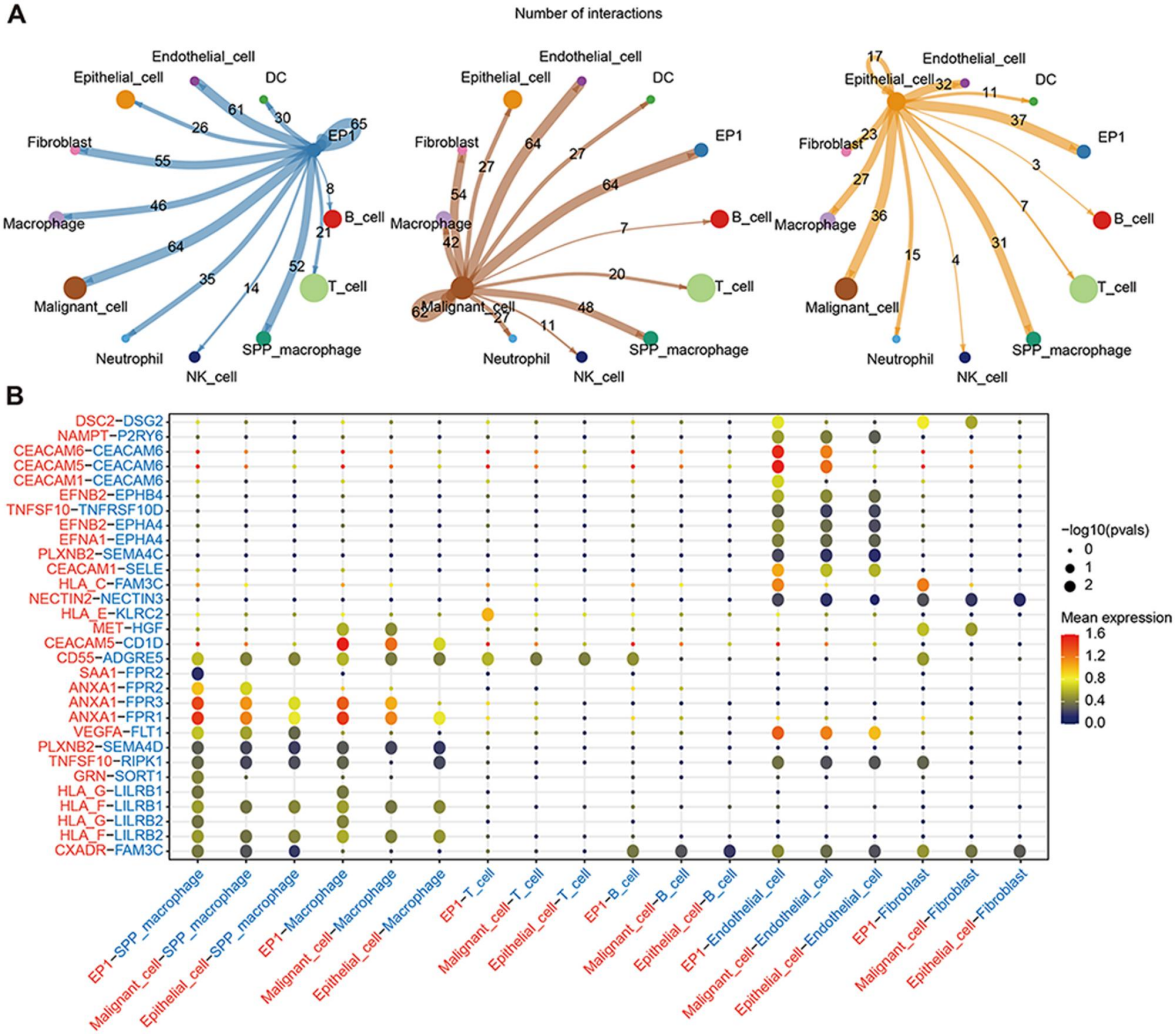
Cell–cell communication network in metastatic CRC. (A) The communication network between EP1 (left), malignant cell (mid), epithelial cell (right), and other types of cells. (B) Dot plot showed ligand-receptor pairs between EP1, malignant, epithelial cell, and other type cell.

To explore the cell–cell interactions of EP1, we performed cell–cell communication analyses based on the CellPhoneDB database. Results showed, compared with normal epithelial cells, EP1 and malignant tumor cells had stronger cell–cell interactions, including a stronger interaction between endothelial cells and SPP^+^ macrophages (Figure 4A). EP1 interacted with endothelial cells by CEA-related cell adhesion molecule (*CEACAM1*, *CEACAM5*, *CEACAM6*; Figure 4B). A stronger interaction between *ANAX1* and formyl peptide receptors (*FPR1*, *FPR2*, *FPR3*) was shown in EP1 and *SPP*^+^ macrophage (Figure 4B). The major histocompatibility complex (*HLA-F*, *HLA-G*) also had a stronger interaction with leukocyte immunoglobulin-like receptor (*LILRB1*, *LILRB2*) between EP1 and *SPP*^+^ macrophage (Figure 4B). These results indicated the stronger interactions between the EP1 and *SPP*^+^ macrophage could lead *SPP*^+^ macrophage to an anti-inflammatory and immunosuppressive phenotype, which may facilitate the tumor metastasis.

### Spatial colocalization between the EP1 subpopulation and *SPP*^+^ macrophage

**Figure 5.**
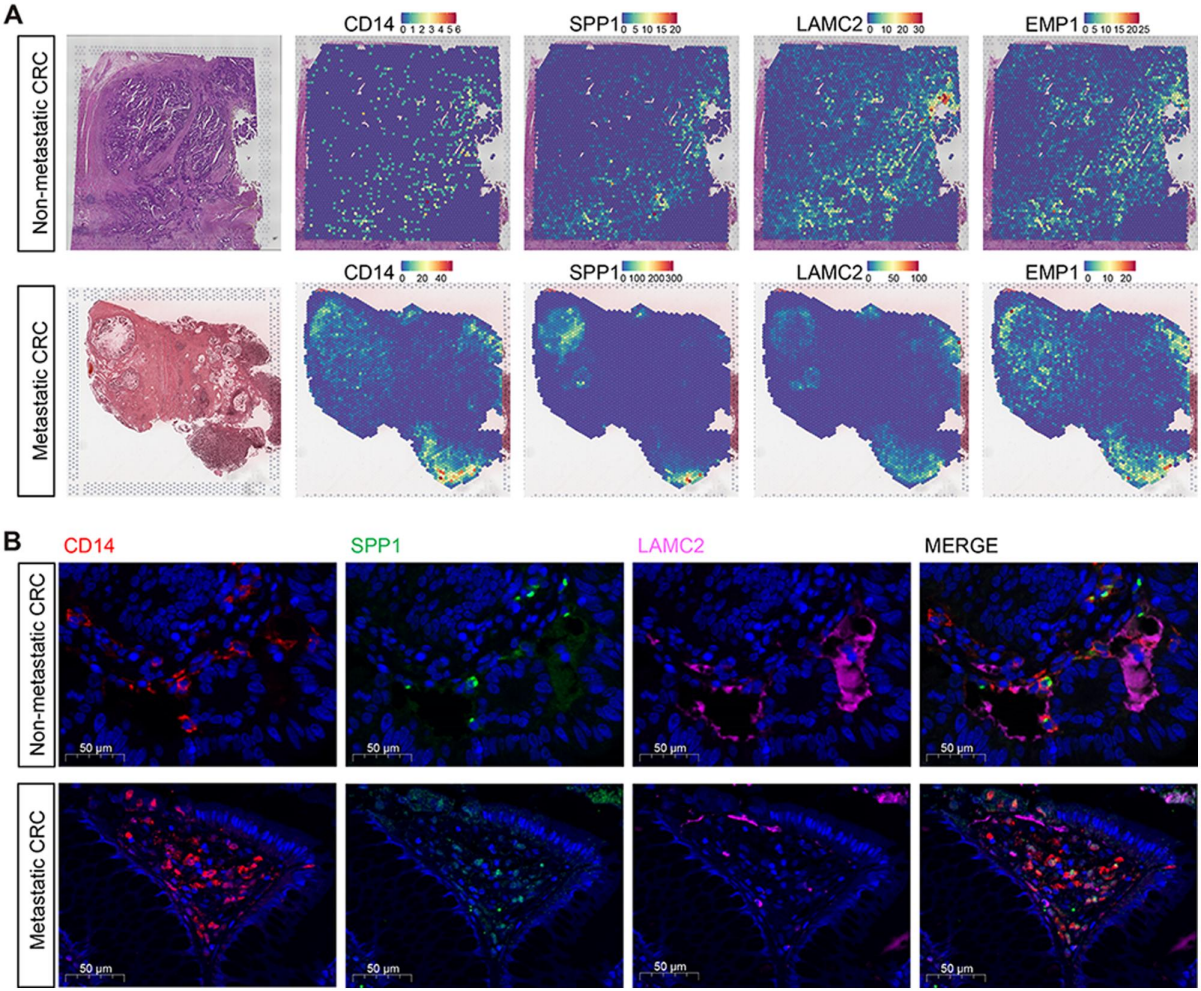
Spatial distribution of EP1 and SPP1+ macrophage. (A) Spatial feature plots showed distribution of *CD14*, *SPP1*, *LAMC2*, and *EMP1* in primary tumor tissues of non-metastatic and metastatic CRC patients. (B) Immunofluorescence staining of *CD14*, *SPP1*, and *LAMC2* in primary tumor tissues of non-metastatic and metastatic CRC patients. Scale bars, 50 μm.

EP1 and *SPP*^+^ macrophage had shown significant cell–cell interactions. To validate the cellular interaction in the spatial level, we analyzed the spatial transcriptomics of metastatic and non-metastatic CRC. Results showed that the marker genes (*KRT17*, *LAMC2*, and *EMP1*) of EP1 were spatially co-expressed with *SPP*^+^ macrophage (Figure 5A). For further validation, we performed an immunofluorescence analysis in primary tumor tissues of metastatic and non-metastatic CRC patients. Similarly, EP1 was co-localized with *SPP*^+^ macrophage (Figure 5B). These results demonstrated the spatial colocalization of EP1 and *SPP*^+^ macrophage.

### Evolutionary characteristics of the metastatic CRC tumor cells

**Figure 6.**
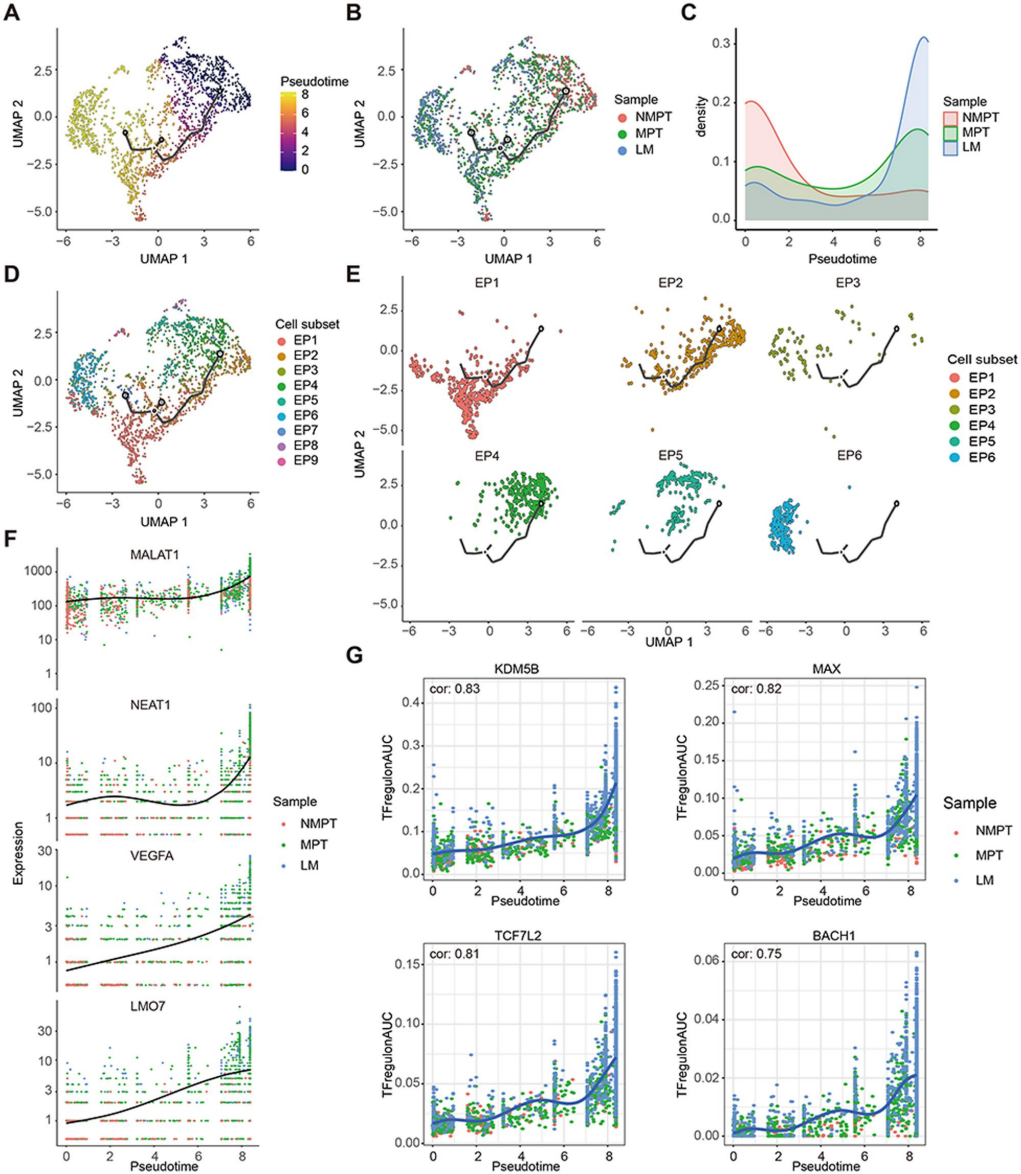
Trajectory analysis of tumor cells in metastatic CRC. (A). Pseudotime analysis of tumor cells. Pseudotime analysis (B) and density plot (C) showed distribution of different samples along the trajectory. Pseudotime analysis (D) and split plot (E) showed distribution of different cell subsets along the trajectory. (F) Scatterplots showed expression levels of genes *MALAT1*, *NEAT1*, *VEGFA*, and *LMO7* along the trajectory. (G) Scatterplots showed area under the curve (AUC) scores of transcription factor (TF) regulons *KDM5B*, *MAX*, *TCF7L2*, and *BACH1* along the trajectory.

To explore the evolutionary characteristics of the metastatic CRC tumor cells, pseudotime trajectory analysis was performed based on malignant tumor cells (NMPT, MPT, and LM; Figure 6A). Results showed that NMPT was in the initial stage, MPT was in an intermediate state, and LM was in a terminal state (Figure 6B, C). This result revealed that tumor cells undergo a phenotypic shift from non-metastatic phenotype to metastatic phenotype during CRC metastasis. Furthermore, analysis of different cell subsets during evolution showed that EP4 and EP5 were in the initial stage, and the proportion of EP1 increased gradually, while the proportion of EP2 decreased gradually (Figure 6D, E), and EP6 was in a terminal state. To find possible targets for blocking tumor metastasis, we explored dynamic changes in genes and TF regulons during evolution. Results showed that the expression level of *MALAT1*, *NEAT1*, *VEGFA*, and *LMO7* increased gradually (Figure 6F, Supplementary Figure S3A), and TF regulons *KDM5B*, *MAX*, *TCF7L2*, and *BACH1* increased gradually during the evolution process (Figure 6G, Supplementary Figure S3B).

## Discussion

Metastasis is the leading cause of CRC mortality. Analyzing the tumor metastatic characteristics, seeking potential therapeutic strategies, and developing prediction models for CRC metastasis are helpful for improving CRC prognosis. In this study, we identified metastasis-related cancer cell subset EP1. We also found EP1 interacted with *SPP*^+^ macrophages and thereby promoted metastasis. Furthermore, we revealed the characteristics of tumor cells from non-metastatic phenotype to metastatic phenotype during the evolution process.

Metastasis-related cancer cell subset EP1 was characterized by gene expression of *KRT17*, *LAMC2*, and *EMP1*. The expression of *KRT17* was significantly associated with shorter disease-free survival time in patients with stage II CRC, suggesting regulation of *KRT17* was linked to tumor metastasis and recurrence [18]. Previous studies have demonstrated *LAMC2* or *EMP1*-expressed cells in tumors. *LAMC2*-expressed cells exhibit a higher migration and invasion ability in pancreatic cancer [19]. CRC cells with a high expression of *EMP1* can be more aggressive and cause tumor metastasis and recurrence in CRC patients [20]. In this study, we found that the *LAMC2*-expressed cells and *EMP1*-expressed cells may be the same cell subset named EP1, which initiates tumor metastasis.

The functional enrichment analysis of EP1 indicated an enhanced cell–cell communication, suggesting that EP1 may facilitate malignant cell metastasis by interacting with the surrounding cells. By analyzing the communication between the EP1 and other cells, we found an enhanced connection between EP1 and vascular endothelial cells and *SPP*^+^ macrophages. EP1 interacted with vascular endothelium through *CEACAM*. *CEACAM* is an adhesion molecule and one of the most widely used tumor biomarkers. *CEACAM* has been used to screen for CRC, and an increased postoperative serum level of CEA usually indicates metastasis and recurrence of CRC [21]. However, the role of *CEACAM* in tumor cells and vascular endothelium is not well defined. Our work demonstrated that *CEACAM* can promote tumor cell adhesion to endothelial cells and favor transendothelial migration. As one of the newly identified subsets of macrophages, *SPP*^+^ macrophages facilitate tumor cell migration and metastasis [14, 15]. We found an enhanced intercellular interaction between the EP1 and *SPP*^+^ macrophages. Spatial transcriptomics and immunofluorescence further showed spatial colocalization between EP1 and SPP+ macrophages. The ligand–receptor interactions analysis showed that the EP1 promoted the *SPP*^+^ macrophage toward an anti-inflammatory phenotype through *ANAX1*-*FRR* [22]. EP1 also transmitted an immune-suppressive signal to *SPP*^+^ macrophages by HLA molecules and leukocyte immunoglobulin-like receptor [23]. Current research demonstrates anti-inflammatory and immunosuppressive tumor-associated macrophages in the tumor microenvironment can promote metastasis [24, 25]. Our study illustrates that EP1 promotes the anti-inflammatory and immunosuppressive phenotype of *SPP*^+^ macrophages, thereby contributing to tumor metastasis.

Our study revealed the dynamic change of tumor cell from non-metastatic to metastatic phenotype during the metastatic process. We also found genes and TF regulons that may be possible therapeutic targets for blocking the tumor metastasis. The expression of *MALAT1*, *NEAT1*, *VEGFA*, and *LMO7* increased gradually in the metastatic process. *MALAT1* is a metastasis-related lncRNA that was first found in non–small cell lung cancer; it can promote tumor invasion and metastasis by facilitating epithelial-to-mesenchymal transition and inducing angiogenic factors [26–28]. Targeting *MALAT1* would inhibit metastasis in multiple tumors [29, 30]. *NEAT1* can activate the Wnt signaling pathway, which could promote CRC progression and metastasis [31, 32]. *VEGFA* is involved in tumor angiogenesis and metastasis, and *VEGFA* inhibitor bevacizumab has been approved by FDA for the targeted therapy of CRC [33, 34]. In addition, *LMO7* has been discovered to regulate tumor metastasis [35–37]. TF regulons *KDM5B*, *MAX*, *TCF7L2*, and *BACH1* gradually increased during the evolution. *KDM5B* is associated with histone methylation and facilitates continuous tumor growth. Research show *KDM5* inhibitors (KDM5i, CPI-455) combined with DAC can reduce breast cancer proliferation *In vitro* [38, 39]. MAX participates in the Myc-Max-Mad transcription factor network and plays a role in cell proliferation, differentiation, and apoptosis [40]. *TCF7L2* can promote gastric cancer metastasis by regulating *PLAUR* [41] and forming a *MIR100HG*/*hnRNPA2B1*/*TCF7L2* axis to promote CRC metastasis [42]. *BACH1* is able to activate the transcription of Hexokinase 2 and *GAPDH* and thereby promotes lung cancer metastasis [43]. These results could suggest new targets for CRC metastasis prevention and prognosis.

There are also some limitations in this study. First, data of circulating tumor cells in metastatic CRC patients are needed to perfect this study. Second, the role of metastasis-related subset EP1 in CRC should be studied by more fundamental experiments.

In conclusion, this study advanced our understanding of CRC metastasis. Based on integrated analysis of CRC metastasis, we found potential therapeutic targets for the prevention and prediction of CRC metastasis. In brief, this study can provide new ideas for the treatment of CRC metastasis and call for more fundamental research in metastasis.

## Supplementary Material

goae055_Supplementary_Data
