# Supplementary material for: Integrated analysis of colorectal cancer metastasis identifies characteristics of tumor cell during metastasis
Source: Gastroenterol Rep (Oxf). 2024 May 30;12:goae055. doi: 10.1093/gastro/goae055 (PMC11139507; doi:10.1093/gastro/goae055)
Supplement: goae055_Supplementary_Data [file goae055_supplementary_data.zip › Table S1.docx]

**Table S1. Clinical information of CRC patients**

| **Patient number** | **Age (years)** | **Gender** | **Cancer location** | **Metastasis** | **Stage** | **Preoperative chemotherapy** |
| --- | --- | --- | --- | --- | --- | --- |
| P1 | 63 | female | ascending colon | no | Ⅱ | no |
| P2 | 51 | male | ascending colon | no | Ⅱ | no |
| P3 | 62 | male | rectum | no | Ⅱ | no |
| P4 | 55 | female | Ascending colon | liver | Ⅳ | no |
| P5 | 45 | male | ascending colon | liver | Ⅳ | no |
| P6 | 73 | male | rectum | liver | Ⅳ | no |
