# Supplementary material for: Integrated analysis of colorectal cancer metastasis identifies characteristics of tumor cell during metastasis
Source: Gastroenterol Rep (Oxf). 2024 May 30;12:goae055. doi: 10.1093/gastro/goae055 (PMC11139507; doi:10.1093/gastro/goae055)
Supplement: goae055_Supplementary_Data [file goae055_supplementary_data.zip › Table S2.docx]

**Table S2. Primer sequences for quantitative real-time polymerase chain reaction**

| **Primer name** | **Primer sequence (from 5’ to 3’)** |
| --- | --- |
| *LAMC2* Forward | TGCATCTGATGGACCAGCCT |
| *LAMC2* Reverse | CCCTCTCTTCCAGCTCTGAC |
| *EMP1* Forward | GCTGGGACCCTTCAGAACTC |
| *EMP1* Reverse | ACCAGCCAGCAATACCAACA |
| *GAPDH* Forward | TGCACCACCAACTGCTTAGC |
| *GAPDH* Reverse | GGCATGGACTGTGGTCATGAG |
