# Supplementary figures and images for: Integrated analysis of colorectal cancer metastasis identifies characteristics of tumor cell during metastasis
Source: Gastroenterol Rep (Oxf). 2024 May 30;12:goae055. doi: 10.1093/gastro/goae055 (PMC11139507; doi:10.1093/gastro/goae055)

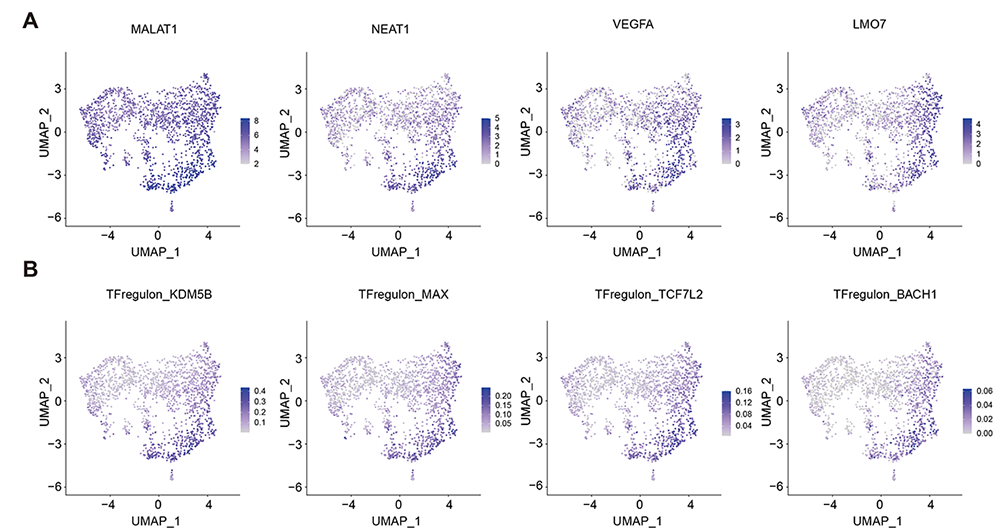

Supplement: goae055_Supplementary_Data [file goae055_supplementary_data.zip › Figure S3.tif]

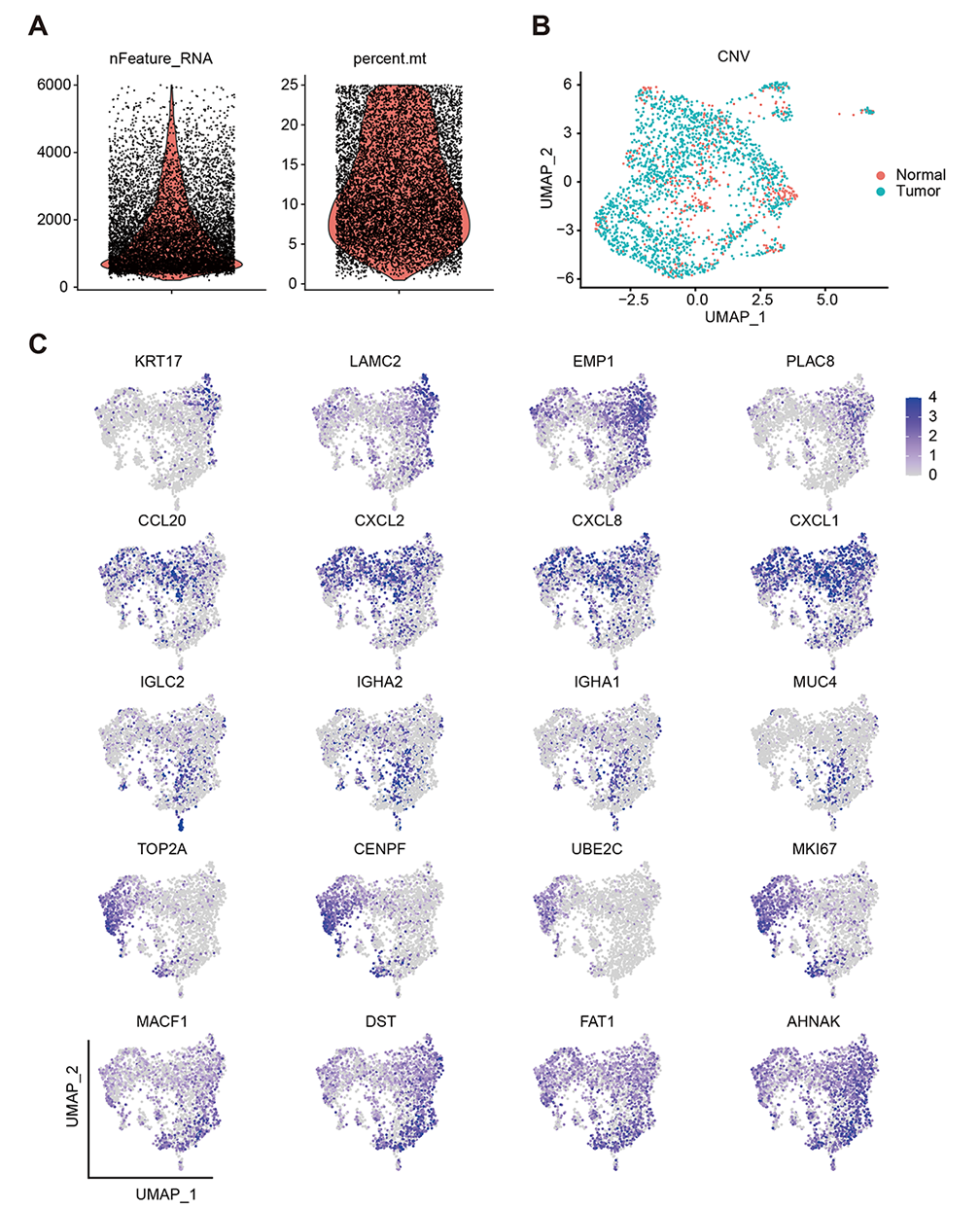

Supplement: goae055_Supplementary_Data [file goae055_supplementary_data.zip › Figure S1.tif]

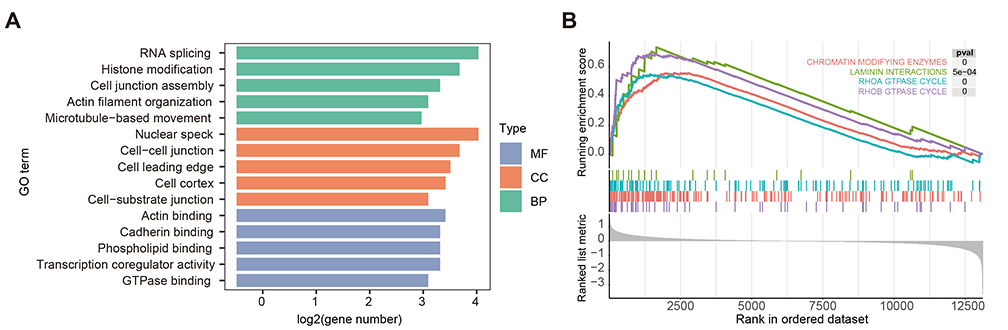

Supplement: goae055_Supplementary_Data [file goae055_supplementary_data.zip › Figure S2.tif]
